# Minimally invasive valve surgery including patients of combined simultaneous surgery: a retrospective study

**DOI:** 10.1186/s13019-023-02361-8

**Published:** 2023-09-30

**Authors:** Yun Ling, Huaxin Chen, Pengxiong Zhu, Tian Li, Bangde Xue, Jun Liu

**Affiliations:** 1 Department of Cardiovascular Surgery, Shanghai East Hospital affiliated to Tongji University School of Medicine, 150 Jimo Rd, Shanghai, 200000 China; 2 Department of Anesthesiology, Hainan Hospital of Chinese PLA General Hospital, 80 Jianglin Rd, Sanya, 572013 China; 3 School of Basic Medicine, Fourth Military Medical University, Xi’an, 710032 China

**Keywords:** Heart valve diseases, Minimally invasive surgical procedures, Cardiac surgical procedures

## Abstract

**Objective:**

This study investigated the perioperative safety and advantages of performing a minimally invasive valve surgery (MIVS) and conducting a preliminary examination of the combined simultaneous surgery (CSS).

**Methods:**

A total of 29 patients (16 men and 13 women; mean age, 58.41 ± 13.08 years) who underwent MIVS at our center from July 2021 to March 2022 were selected. Among them, 16 patients underwent aortic valve surgery (AVS), 13 patients underwent mitral valve surgery (MVS), and four patients additionally underwent CSS.

**Results:**

The MIVS time ranged from 165 to 420 min, with a mean of 230.54 ± 54.61 min; the cardiopulmonary bypass (CPB) time ranged from 54 to 164 min, with a mean of 120.24 ± 25.98 min; the aortic cross-clamp (ACC) time ranged from 36 to 118 min, with a mean of 78.66 ± 21.01 min and an automatic heart resuscitating rate was 89.66%; the mean tracheal intubation time was 6.30 ± 3.87 h, and the median total postoperative drainage was 317.5 (35, 1470) ml. No difference was observed between preoperative and postoperative left ventricular ejection fraction (LVEF) (61.90% ± 6.28% vs. 60.21% ± 5.52%, *P* = 0.281). The difference in postoperative drainage (419.20 ml ± 377.20 ml vs. 588.75 ml ± 673.63 ml, *P* = .461), tracheal intubation time (6.66 h ± 4.27 h vs. 4.63 h ± 1.11 h, *P* = .359), intensive care unit (ICU) stay (3.96 ± 8.62 days vs. 2.00 ± 0.816 days, *P* = .658), and postoperative hospital stay (9.96 ± 8.45 days vs. 8.25 ± 1.26 days, *P* = .694) between MIVS and CSS was not significant.

**Conclusion:**

MIVS in our center may be safe and effective. Additionally, CSS may be a feasible option that could be performed after a thorough preoperative evaluation and multidisciplinary discussion.

## Introduction

With the advancements in cardiac surgery-related technologies, minimally invasive heart valve surgery has recently become a center of attention. Furthermore, the advent of interventional valve-therapy techniques, like transcatheter aortic valve implantation (TAVI) and MitraClip™ has had a considerable impact on the evolution of open-heart valve surgery. However, interventional therapies, like TAVI, MitraClip™, etc. still have pertinent surgical limitations.

The choice of valve material is a significant limitation for TAVI. Presently, TAVI is recommended for patients older than or equal to 65 years with significant surgical risk factors [1]. However, patients with left ventricular thrombosis, left ventricular outflow tract obstruction, interventional access, or aortic root morphology are not suitable for TAVI. Moreover, TAVI is contraindicated in patients with a life expectancy lesser than 12 months after AS correction [2–5].

Similarly, current clinical catheter-based edge-to-edge technology using MitraClip™ applies selectively to patients with appropriate mitral valve anatomy, left ventricular ejection fraction (LVEF) between 20 and 50%, left ventricular end-systolic dimension (LVESD) less than or equal to 70 mm, and pulmonary artery systolic pressure (PASP) less than or equal to 70 mmHg [6–10].

To summarize, open-heart surgery cannot be entirely replaced shortly. Hence, it is presently crucial to reduce surgical trauma, shorten the surgical time, and decrease the incidence of complications [11]. We studied minimally invasive valve surgery (MIVS) and possibility to combined simultaneous surgery (CSS) at our hospital from July 2021 to January 2022. These findings are summarized and analyzed in this article.

## Material and methods

### General information

Subjects satisfying the following criteria were included in this study: (1) Patients with heart valve disorders, including aortic and mitral valvular diseases, with cardiac function in class II–III (NYHA functional classification), and FEV1 ≥ 50%; (2) Patients having no history of thoracic surgery; and (3) eGFR ≥ 60 ml/min and Child–Pugh grade A. We excluded patients with double valve lesions or those with complex cardiac pathology. Preoperative diagnosis was made primarily by color Doppler echocardiography, electrocardiography, chest CT, and coronary angiography whom with CHD Risk Factors. A total of 29 patients underwent MIVS and their general information is presented in Table 1.

**Table 1:** Basic information of patients (x ± s)

Variables	Values
Sex (man/woman)	16/13
Age (years)	58.41 ± 13.08
Height (cm)	166.62 ± 8.42
Body weight (kg)	63.86 ± 12.69
Cardiac function	
Class II (%)	7 (24.1)
Class III (%)	22 (75.9)
Hypertension (%)	16 (55.2)
Type 2 diabetes (%)	3 (10.3)
Hyperlipidemia (%)	1 (3.4)
History of smoking (%)	8 (27.6)
Chronic alcohol consumption (%)	8 (27.6)

### Surgical method

MIVS was performed with cardiopulmonary bypass (CPB). The patient's right side was elevated at 45° to place the external defibrillation electrodes. The electrode plates were placed behind the right scapula and between the fifth and sixth ribs from the left anterior axillary line. Preoperatively, the anesthesiologist prepared a guidewire by puncturing the left internal jugular vein. The femoral arterial and the femoral venous cannulas were used as arterial perfusion and inferior vena cava drainage tubes, respectively. Intraoperative trans-esophageal echocardiography (TEE) was performed to observe the position of the femoral venous cannula. The cannula opening was placed at the superior vena cava opening. Intraoperative venous drainage was done to increase the negative pressure appropriately. In the case of unsatisfactory intraoperative drainage, a superior vena cava drainage tube was placed via a left internal jugular vein guidewire to ensure adequate drainage.

**Fig. 1:**
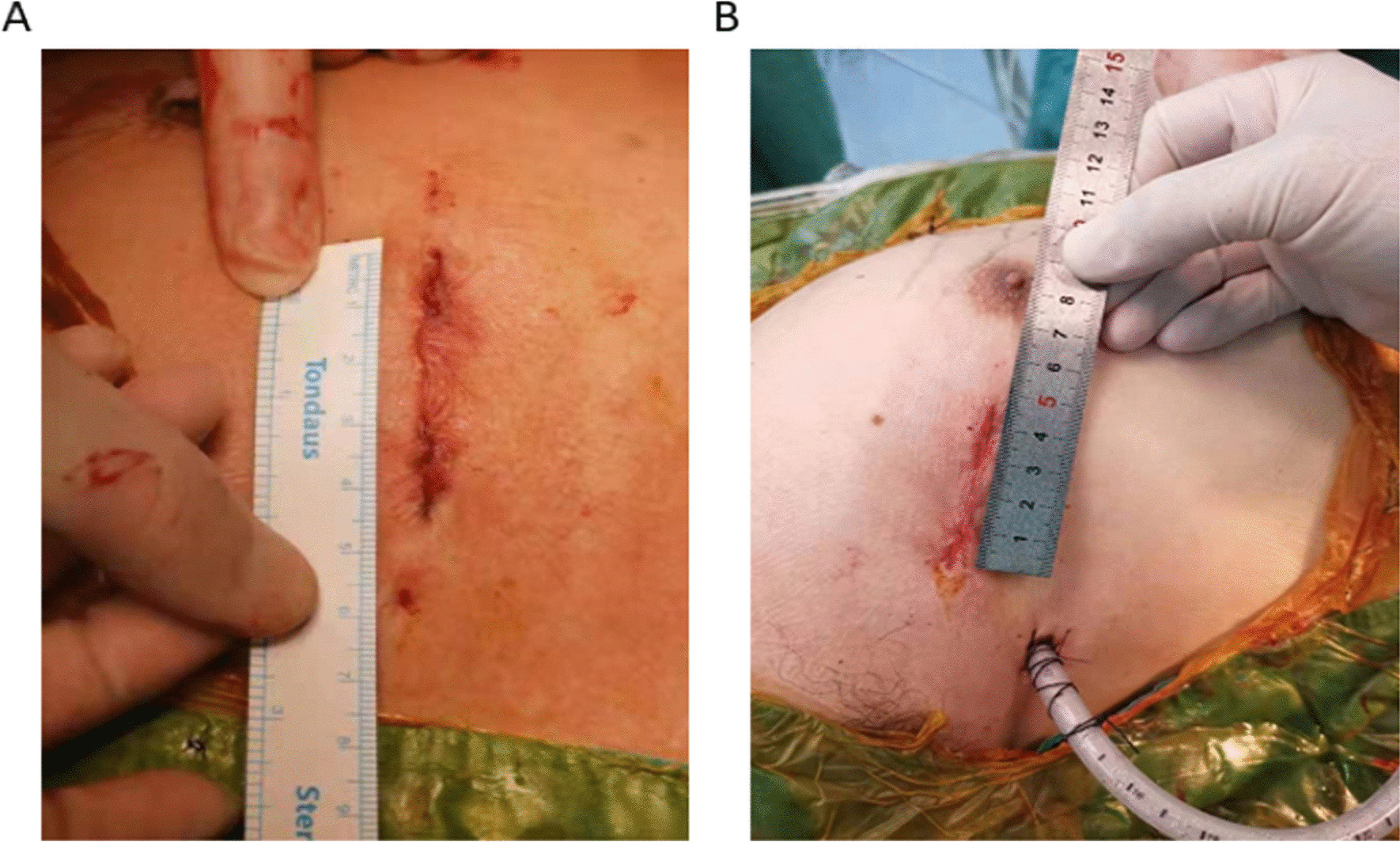
Incisions after AVS and MVS. **A** Incision closed after AVS. **B** Incision closed after MVS

**Fig. 2:**
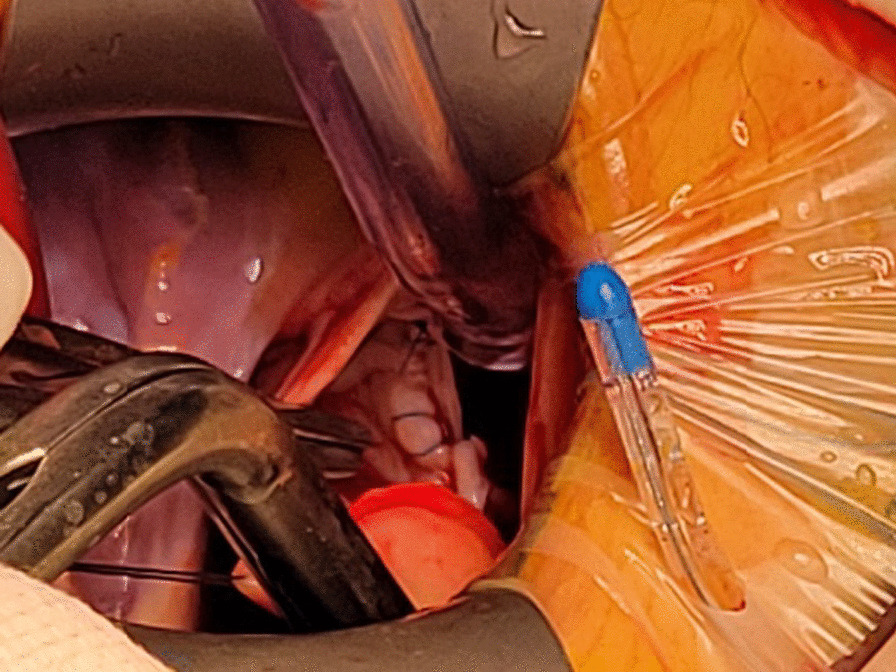
Low-flow carbon dioxide was used to perfuse the surgical field

Small-incision aortic valve surgery (AVS) was performed with an arcuate incision in the second intercostal space on the right side of the sternum (approximately 4–5 cm) (Fig. 1A). Additionally, small-incision mitral valve surgery (MVS) was performed with an arcuate incision in the anterior axillary line of the fourth intercostal space on the right side of the chest (4–5 cm) (Fig. 1B) to expose the mitral valve by an interatrial-sulcus-left-atrial incision. The ascending aorta was cross-clamped and Del Nido myocardial protective fluid (1000 ml) was perfused in an anterograde manner through the ascending aorta. In case of aortic cross-clamp time exceeding 90 min, another anterograde perfusion was done. Before giving an incision over the cardiac region, continuous low-flow carbon dioxide was used to perfuse the surgical field (Fig. 2) until the cardiac incisions were closed. The carbon dioxide flow was turned off after the aortic cross-clamping was opened and continuous negative pressure suction exhaustion was completed.

### Observational indicators

Observational indicators included the surgical time, cardiopulmonary bypass time (CPB time), aortic cross-clamp (ACC) time, automatic heart resuscitating rate, intensive care unit (ICU) stay, postoperative hospital stay, total postoperative drainage, and changes in left atrial diameter (LAD), left ventricular end-diastolic dimension (LVEDD), and left ventricular ejection fraction (LVEF) one week after surgery.

### Statistical analysis

SPSS23 software was used for statistical analyses. Measurement data were expressed as mean ± standard deviation and analyzed using an independent-samples T-test. Enumeration data were analyzed using the chi-square test. *P* < 0.05 indicated a significant difference.

## Results

### Overall results

Among the 29 patients, 14 underwent aortic valve replacement, one underwent aortic valve replacement with coronary artery bypass grafting (the target artery was right coronary artery, and the used conduits was saphenous vein), one underwent aortic valvuloplasty, 10 underwent mitral valvuloplasty, and one underwent mitral valvuloplasty with auricular septal defect repair, and two underwent mitral valve replacement (Table 2). The surgical time was 165–420 min, with an average of 230.54 ± 54.61 min; the CPB time was 54–164 min, with an average of 120.24 ± 25.98 min; the ACC time was 36–118 min, with an average of 78.66 ± 21.01 min; the automatic heart resuscitating rate was 89.66%; the mean tracheal intubation time was 6.30 ± 3.87 h; the median total postoperative drainage was 317.5 (35, 1470) ml. No postoperative hepatic or renal insufficiency and perioperative neurological complications occurred in all patients. One patient died in the perioperative period. Additionally, one patient underwent reintubation after postoperative extubation. One patient also underwent emergency surgery for acute infective endocarditis.

**Table 2:** Intraoperative and postoperative indicators

Variables	Values
Operation time (min)	230.54 ± 54.61
Cardiopulmonary bypass time (min)	120.24 ± 25.98
Aortic cross-clamp time (min)	78.66 ± 21.01
Automatic heart resuscitating rate	26 (89.66)
Postoperative tracheal extubation time	6.30 ± 3.87
ICU stay (d)	3.84 ± 8.63
Postoperative hospitalization days (d)	9.71 ± 8.14
Total postoperative drainage (ml)	317.5 (35, 1470)

Significant differences were noted between preoperative and postoperative LAD (*P* = 0.026) in MVS and LVEDD (*P* < 0.001) in AVS on echocardiography. Furthermore, there was a significant reduction in the postoperative cardiac size as compared to the preoperative one. (Table 3).

**Table 3:** Comparison of preoperative and postoperative echocardiographic indicators

Indicators	Preoperative value	Postoperative value	*P* value
Left atrial diameter (mm)	40.80 ± 6.95	37.26 ± 4.63	0.026
Left ventricular end-diastolic diameter (mm)	52.72 ± 6.88	46.64 ± 5.08	< 0.001
Left ventricular ejection fraction (%)	61.90 ± 6.28	60.21 ± 5.52	0.281

### Analysis of AVS and MVS subgroups

We further compared the preoperative and postoperative echocardiographic indicators following AVS and MVS, respectively. After AVS, there was no significant difference recorded between preoperative and postoperative LAD (*P* = 0.291) and LVEF (*P* = 0.803), whereas a significant difference was recorded in LVEDD (*P* = 0.003) (Table 4). After MVS, a significant difference between preoperative and postoperative LAD (*P* = 0.009) was observed. Additionally, a difference in LVEDD was also recorded, but it was not statistically significant (*P* = 0.045). Furthermore, there was a reduction in preoperative LVEF as compared to the postoperative values, but the difference was not significant (Table 5).

**Table 4:** Comparison of echocardiographic indicators before and after aortic valve surgery

Indicators	Preoperative value	Postoperative value	*P* value
Left atrial diameter (mm)	38.06 ± 5.88	36.08 ± 4.43	0.291
Left ventricular end-diastolic diameter (mm)	53.50 ± 7.60	46.46 ± 4.50	0.003
Left ventricular ejection fraction (%)	60.19 ± 6.90	59.63 ± 5.62	0.803

**Table 5:** Comparison of echocardiographic indicators before and after mitral valve surgery

Indicators	Preoperative value	Postoperative value	*P* value
Left atrial diameter (mm)	45.46 ± 5.50	39.92 ± 4.38	0.009
Left ventricular end-diastolic diameter (mm)	51.77 ± 6.03	46.83 ± 5.90	0.045
Left ventricular ejection fraction (%)	64.00 ± 4.90	60.92 ± 5.53	0.146

### CSS

Of the 29 patients who participated in this study, four additionally underwent CSS. The following surgeries were performed on these four patients, respectively: small-incision aortic valvuloplasty with simultaneous radical resection of the thyroid due to cancer, small-incision aortic valve replacement with simultaneous repair of the umbilical hernia, small-incision aortic valve replacement with simultaneous catheter ablation of atrial fibrillation, and small-incision mitral valvuloplasty with simultaneous laparoscopic right adrenal tumor resection and microscopic left ureter holmium laser lithotripsy. The Postoperative drainage of CSS were 588.75 ± 673.63 mL and tracheal intubation time were 4.63 ± 1.11 h. ICU stay of CSS were 2.00 ± 0.816 days and postoperative hospitalization were 8.25 ± 1.26 days (Table 6).

**Table 6:** Comparison and valve surgery alone and combined simultaneous surgery

Variables	Without simultaneous surgery	With simultaneous surgery	*P* value
Postoperative drainage	419.20 ± 377.20	588.75 ± 673.63	0.461
Tracheal intubation time	6.66 ± 4.27	4.63 ± 1.11	0.359
ICU stay	3.96 ± 8.62	2.00 ± 0.816	0.658
Postoperative hospitalization days	9.96 ± 8.45	8.25 ± 1.26	0.694

## Discussion

**Fig. 3:**
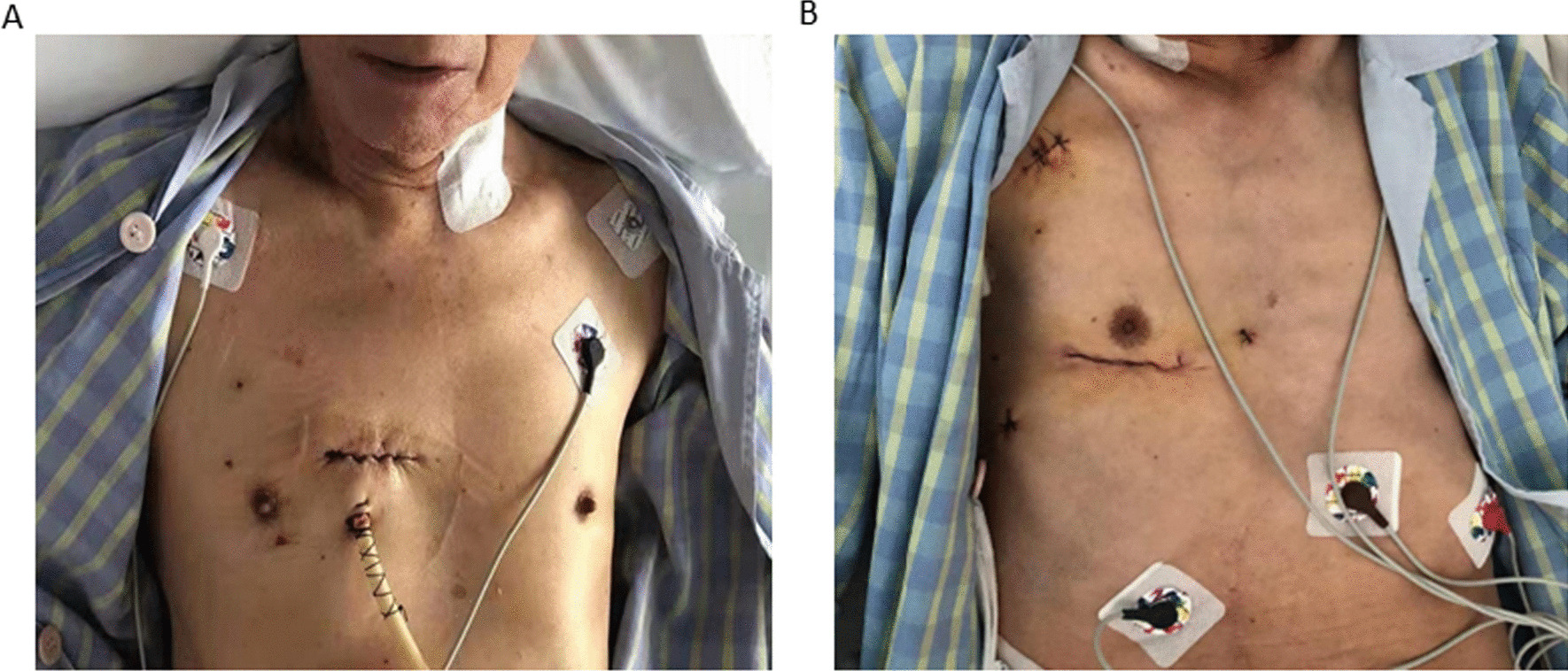
The incisions of AVS and MVS before discharge. **A** AVS. **B** MVS

Open-heart surgery has a history of nearly 70 years and is technically mature [12]. However, in most open-heart surgeries, a median sternotomy incision occurs, which leads to severe trauma, a long sternal healing time, and extremely high mortality in the event of mediastinal infection. The whole treatment process of mediastinal infection greatly increases the patients’ pain. In this study, all 29 valve surgeries were performed with a small incision in the right intercostal space, without breaking the ribs and damaging the sternum. Thus, the integrity of the entire thorax was maintained and postoperative sternal complications were avoided [13]. The small incision also provides better cosmetic outcomes and patient acceptance [14, 15] (Fig. 3).

Intraoperative data revealed that the surgical time (253.94 ± 61.43 min for AVS; 204.38 ± 28.47 min for MVS), ACC time (87.63 ± 22.30 min for AVS; 67.62 ± 13.02 min for MVS), and CPB time (128.50 ± 28.93 min for AVS; 110.08 ± 18.08 min for MVS) of both AVS and MVS were similar to other reports [16–18], with an automatic heart resuscitating rate of 89.66%. Also, postoperative hepatic or renal insufficiency was not seen in all patients. The intraoperative myocardial protection and organ perfusion were good. Intraoperative TEE that was routinely performed in each case to assess the surgical outcomes, indicated an absence of perivalvular leakages. One study suggested that femoral arterial cannulation with perfusion increases the risk of perioperative stroke and delirium [19]. Presently, it is accepted that the risk of neurological complications is similar in conventional median sternotomy incision-based valve surgery and intercostal small-incision-based valve surgery [20]. Furthermore, perioperative neurological complications were absent in all patients in this study.

Further comparison between preoperative and postoperative echocardiographic indicators of AVS and MVS suggested a significant decrease in LVEDD and cardiac preload within one week after AVS. LAD changed more significantly than LVEDD after MVS, in the first week. This was consistent with the presentation of corrected mitral valve diseases and indicated a good surgical outcome in the first week. The reduction of the ejection fraction in the postoperative period of patients with mitral regurgitation is insignificant as it is presumably overestimated in the preoperative period due to the pathology.

Of the 29 patients, one died of severe postoperative pulmonary infection resulting in septicemia and infectious shock. One patient was reintubated after extubation of oral intubation due to postoperative pulmonary infection. The cause of pulmonary infection needs further investigation as it is currently unclear if it is associated with preoperative respiratory function assessment, intraoperative one-lung ventilation, or postoperative painful intercostal incision causing difficulty in sputum expectoration.

In our study, four patients underwent CSS with minimally invasive heart valve surgery, involving two cases of general surgery, one case of cardiology, and one case of urology. We fully evaluated the extent of valve lesions and surgical indications of the patients before surgery and conducted multidisciplinary discussions by including experts from surgery, anesthesiology, critical care medicine, and internal medicine departments. Furthermore, the sequence of cardiac surgery and CSS, possible accidents during anesthesia, intraoperative positional changes, and postoperative management were fully discussed to establish contingency plans. The advantages of CSS were as follows: (1) anticoagulation and antiplatelet therapy are required after heart valve surgery while staging surgery requires discontinuation of anticoagulation, which increases the risk of thromboembolism; (2) It can avoid delays in surgery for other diseases by surgical staging; (3) It can avoid repeated admissions, examinations, and reduce the frequency of anesthesia, thus providing more benefits to patients. All four patients in this study recovered well after surgery. Additionally, there was no significant difference in the total postoperative drainage, tracheal intubation time, ICU stay, and postoperative hospitalization stay of patients who underwent additional CSS compared to those who underwent cardiac surgery alone. Despite the small sample size and insufficient data, the good outcomes of the four patients provide a base for conducting further clinical evaluation and discovering newer treatment options in patients who require multiple surgeries.

However, this study has the following limitations: (1) a short follow-up period, hence, further follow-up is required to evaluate surgical outcomes and prognosis; (2) postoperative drainage volume and tracheal intubation time and other in-significant comparisons may be affected by the small sample size. In the next study, we will further increase the sample size and extend the follow-up time to clarify the long-term effect.

## Data Availability

Raw data are available from corresponding author upon reasonable request.

## Abbreviations

MIVS: Minimally invasive valve surgery
CSS: Combined simultaneous surgery
CPB: Cardiopulmonary bypass
AVS: Aortic valve surgery
MVS: Mitral valve surgery
ACC: Aortic cross-clamp
